# Tramadol for premature ejaculation: a systematic review and meta-analysis

**DOI:** 10.1186/1471-2490-15-6

**Published:** 2015-01-30

**Authors:** Marrissa Martyn-St James, Katy Cooper, Eva Kaltenthaler, Kath Dickinson, Anna Cantrell, Kevan Wylie, Leila Frodsham, Catherine Hood

**Affiliations:** School for Health and Related Research (ScHARR), University of Sheffield, Regent Court, 30 Regent Street, Sheffield, S1 4DA UK; Porterbrook Clinic, Sexual Medicine, Sheffield, UK; Institute of Psychosexual Medicine, London, UK; St George’s Hospital, London, UK

**Keywords:** Premature ejaculation, Tramadol, Systematic review, Meta-analysis, Efficacy, Safety

## Abstract

**Background:**

Tramadol is a centrally acting analgesic prescribed off-label for the treatment of premature ejaculation (PE). However, tramadol may cause addiction and difficulty in breathing and the beneficial effect of tramadol in PE is yet not supported by a high level of evidence. The purpose of this study was to systematically review the evidence from randomised controlled trials (RCT) for tramadol in the management of PE.

**Methods:**

We searched bibliographic databases including MEDLINE to August 2014 for RCTs. The primary outcome was intra-vaginal ejaculatory latency time (IELT). Methodological quality of RCTs was assessed. Between-group differences in IELT and other outcomes were pooled across RCTs in a meta-analysis. Statistical and clinical between-trial heterogeneity was assessed.

**Results:**

A total of eight RCTs that evaluated tramadol against a comparator were included. The majority of RCTs were of unclear methodological quality due to limited reporting. Pooled evidence (four RCTs, 721 participants), suggests that tramadol is significantly more effective than placebo at increasing IELT over eight to 12 weeks (p = 0.0007). However, a high level of statistical heterogeneity is evident (I-squared = 74%). Single RCT evidence indicates that tramadol is significantly more effective than paroxetine taken on-demand, sildenafil, lidocaine gel, or behavioural therapy on IELT in men with PE. Tramadol is associated with significantly more adverse events including: erectile dysfunction, constipation, nausea, headache, somnolence, dry mouth, dizziness, pruritus, and vomiting, than placebo or behavioural therapy over eight to 12 weeks of treatment. However, addiction problems or breathing difficulties reported by patients for PE is not assessed in the current evidence base.

**Conclusions:**

Tramadol appears effective in the treatment of PE. However, these findings should be interpreted with caution given the observed levels of between-trial heterogeneity and the reporting quality of the available evidence. The variability across placebo-controlled trials in terms of the tramadol dose evaluated and the treatment duration does not permit any assessment of a safe and effective minimum daily dose. The long-term effects and side effects, including addiction potential, for men with PE have not been evaluated in the current evidence base.

**Trial registration:**

The review is registered on PROSPERO 2013:CRD42013005289.

**Electronic supplementary material:**

The online version of this article (doi:10.1186/1471-2490-15-6) contains supplementary material, which is available to authorized users.

## Background

Premature ejaculation (PE) is commonly defined by a short ejaculatory latency, a perceived lack of ejaculatory control; both related to self-efficacy; and distress and interpersonal difficulty [1]. PE can be either lifelong (primary), present since first sexual experiences, or acquired (secondary), beginning later [2]. The recently updated International Society of Sexual Medicine’s Guidelines for the Diagnosis and Treatment of Premature Ejaculation (PE) propose that PE is a male sexual dysfunction characterised by ejaculation within about one minute of vaginal penetration (lifelong PE) or a reduction in latency time to ≤3 minutes (secondary PE), the inability to delay ejaculation, and negative personal consequences [3].

The treatment of PE should attempt to alleviate concern about the condition as well as increase sexual satisfaction for the patient and the partner [4]. Available treatment pathways for the condition are varied and treatments may include both behavioural and/or pharmacological interventions. Tramadol is a centrally acting analgesic agent that combines opioid receptor activation and re-uptake inhibition of serotonin and noradrenaline, prescribed off-label for the treatment of PE. Dapoxetine (a selective serotonin re-uptake inhibitor) is currently the only approved oral drug to treat PE. In May 2009, the US Food and Drug Administration released a warning letter about tramadol's potential to cause addiction and difficulty in breathing [5]. Tramadol has previously been evaluated by three systematic reviews [6–8], two of which have pooled data in a meta-analysis [7, 8]. The search methodology and inclusion criteria vary across these reviews. Of the two reviews including a meta-analysis, one [7] pooled data across different study types (observational studies and RCTs) using a mean difference [7]. One review pooled IELT effect estimates across studies using a standardised mean difference [8]. The European Association of Urology guidelines for the management of PE summarise that tramadol has shown a moderate beneficial effect with a similar efficacy as dapoxetine. However, that the beneficial effect of tramadol in PE is yet not supported by a high level of evidence [9].

The aim of this study was to systematically review the evidence base for tramadol in the management of PE, by summarising evidence from randomised controlled trials (RCTs) and reporting a mean difference meta-analysis of RCT IELT data. The review addressed the question “in men with premature ejaculation, what is the clinical effectiveness of tramadol as compared with a non-active comparators or other treatments, evaluated in randomised controlled trials”. The review is registered on PROSPERO 2013:CRD42013005289. Available from http://www.crd.york.ac.uk/PROSPERO/display_record.asp?ID=CRD42013005289.

## Methods

The review was undertaken in accordance with the general principles recommended in the Preferred Reporting Items for Systematic Reviews and Meta-Analyses (PRISMA) statement [10].

### Searches

The following databases were searched from inception to 5 August 2014 for published and unpublished research evidence: MEDLINE; Embase; Cumulative Index to Nursing and Allied Health Literature (CINAHL); The Cochrane Library including the Cochrane Systematic Reviews Database (CDSR), Cochrane Controlled Trials Register (CCRT), Database of Abstracts of Reviews of Effects (DARE) and the Health Technology Assessment (HTA) database; ISI Web of Science (WoS), including Science Citation Index, and the Conference Proceedings Citation Index-Science. Full search terms are reported elsewhere [11]. The U.S. Food and Drug Administration (FDA) website and the European Medicines Agency (EMA) website were also searched. Existing systematic reviews were also checked for eligible studies. All citations were imported into Reference Manager Software and any duplicates deleted. The MEDLINE search strategy is presented as an Additional file 1.

### Study selection

Searches were screened for potentially relevant studies by one reviewer and a subset checked by a second reviewer (and a check for consistency undertaken). Full texts were screened by two reviewers. Details of studies identified for inclusion were extracted using a data extraction sheet.

### Eligible studies

RCTs in adult men with PE that evaluated tramadol were eligible for inclusion. Randomised crossover design studies were excluded to avoid double counting of participants in the meta-analysis. Theses and dissertations were not included. Non-English publications were included where sufficient data could be extracted from an English-language abstract or tables.

### Outcomes

The primary outcome was intra-vaginal ejaculatory latency time (IELT). Other outcomes included sexual satisfaction, control over ejaculation, relationship satisfaction, self-esteem, quality of life, treatment acceptability and adverse events.

### Data extraction

One reviewer performed data extraction of each included study. All numerical data were then checked by a second reviewer.

### Methodological quality of studies

Methodological quality of RCTs was assessed using the Cochrane Collaboration risk of bias assessment criteria [12]. We classified RCTs as being at overall ‘low’ or ‘high’ risk of bias if they were rated as such for each of three key domains - allocation concealment, blinding of outcome assessment and completeness of outcome data (attrition <30%).

### Data synthesis

Where possible, between-group differences for direct comparisons (e.g., tramadol *vs.* placebo) were pooled across trials in a pairwise meta-analysis using Cochrane RevMan software (version 5.2) (RevMan 2012 [13]). Continous variables were analysed as a mean difference (MD) and dichotomous variables as a risk ratio (RR). No subgroup or sensitivity analyses were planned. For comparisons where there was little apparent clinical heterogeneity and the *I*^2^ value (*I*^2^ statistic [14]) was 40% or less, a fixed-effect model was applied. Random-effects models were applied where *I*^2^ value was >40%. Between-group effect estimates were considered significant at p < 0.05. Where >5 RCT comparisons were available, publication bias was assessed by visual inspection of funnel plots.

### Ethical approval and consent from patients

The project was not primary research involving humans or animals but was a secondary analysis of human subject data available in the public domain.

## Results

### Search results

**Figure 1:**
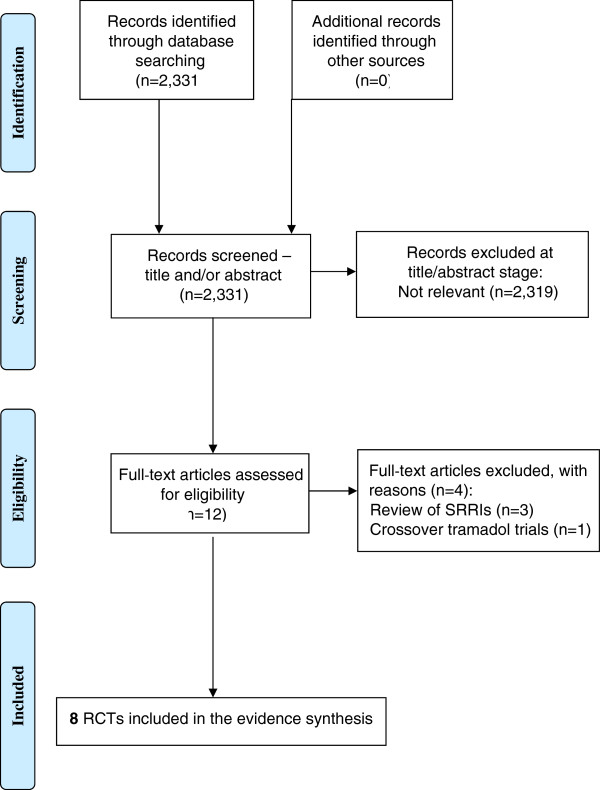
**Study selection process - Preferred Reporting Items for Systematic Reviews and Meta-Analyses (PRISMA) flow diagram.**

The searches identified 2,331 citations (as part of a wider project assessing a variety of treatments for PE [11]). Of these, 2,319 citations were excluded as titles/abstracts. Twelve full-text articles were obtained as potentially relevant. The study selection process is fully detailed in the PRISMA flow diagram in Figure 1. A total of seven RCTs that evaluated tramadol against a comparator (placebo, another agent, or behavioural therapy) and one RCT that evaluated different tramadol doses (eight RCTs in total) were identified.

Details of the included RCTs, the comparator(s), outcomes assessed and the risk of bias assessment are detailed in Table 1.

**Table 1:** **RCT characteristics, efficacy and safety outcomes, and risk of bias assessment**

RCT (country) duration	PE definition, Lifelong/acquired PE, erectile dysfuntion	Treatment, comparator, numbers analysed/randomised (%) When taken	Efficacy outcomes and results	Adverse events	Risk of bias assessment
Alghobary 2010 [15] (Egypt) 6 weeks	DSM-IV-TR All lifelong PE ED, NR	- Tramadol 50 mg 2 to 3 h PC, 17/17 (100%) - Paroxetine 20 mg/d, 18/18 (100%)	IELT (Stopwatch): see Figure 3. Arabic Index of Premature Ejaculation (AIPE): significant improvement in scores at 6 weeks with both tramadol and paroxetine. Difference between groups not significant. Tramadol group had less rigid erections than paroxetine group	The drugs were generally tolerated and no serious side-effects encountered apart from mild headache and gastric upset with paroxetine and mainly gastric upset with tramadol and no withdrawn cases recorded.	Unclear risk - allocation method and blinded outcome assessment not reported
Bar-Or 2012 [18] (11 EU countries) 12 weeks	DSM-IV-TR All lifelong PE ED, excluded	- Tramadol 62 mg, 206/232 (89%) - Tramadol 89 mg, 198/217 (91%) - Placebo, 200/228 (88%) 2 to 8 h PC	IELT (Stopwatch): see Figure 3. Premature Ejaculation Profile (PEP): Mean change for all 4 measures significantly higher in both tramadol groups than placebo Female partner PEP scores: more had improvement (> = 1 category) for tramadol than placebo on all 4 measures	Any adverse event: Tramadol 62 mg: 12% Tramadol 89 mg: 16% Placebo: 7% No difference was observed in the incidence of withdrawal by treatment group (0.0% placebo, 1.0% 62 mg tramadol, 1.6% 89 mg tramadol). There were no serious AEs.	Unclear risk - allocation method and blinded outcome assessment not reported
Eassa 2013[20] (Egypt) 24 weeks	PE def, NR All lifelong PE ED, excluded	- Tramadol 25 mg, 100/100 (100%) - Tramadol 50 mg, 100/100 (100%) - Tramadol 100 mg, 100/100 (100%) 2 to 3 h PC	IELT (Stopwatch): see Figure 3	Tramadol 25 mg - somnolence (100%); pruritus (100%) Tramadol 50 mg - somnolence (100%); pruritus (100%); dizziness (18%); headache (16%); dry mouth (13%) Tramadol 100 mg - somnolence (100%); pruritus (100%); dizziness (38%); headache (30%); dry mouth (20%); nausea (20%); vomiting (17%)	Unclear risk - allocation method and blinded outcome assessment not reported
Gameel 2013 [16] (Egypt) 4 weeks	IELT of <2 min in >75% of episodes All had PE for >1 year ED, excluded	- Tramadol 50 mg 2 h PC + inert lubricating gel 15 min PC, 29/30 (97%) - Sildenafil 50 mg 1 h PC + inert lubricating gel 15 min PC, 30/30 (100%) - Paroxetine 20 mg 4 h PC + inert lubricating gel 15 min PC, 28/30 (93%) - Lidocaine gel 15 min PC + oral multivitamin 1-4 h PC, 30/30 (100%) - Placebo (oral multivitamin 1-4 h PC + inert lubricating gel 15 min PC), 27/30 (90%)	IELT (stopwatch): see Figure 3. Sexual satisfaction (0 to 5 point scale: Tramadol and paroxetine were associated with comparable drug-induced improvements in sexual satisfaction, but tramadol was associated with significantly better sexual satisfaction scores than was the local anaesthetic.	Greater sleep disturbance, dry mouth, nausea, dizziness, fatigue, vomiting, sweating, and headache were reported with tramadol, sildenafil and paroxetine. All side effects were reported as being tolerable.	Unclear risk - allocation method and blinded outcome assessment not reported
Kahn 2013 [21] (India) 8 weeks	DSM-IV TR 41/60 (68%) lifelong PE ED, excluded	- Tramadol 100 mg/d, 4 weeks then 2 or 8 h PC, 4 weeks; 30/30 (100%) - Placebo/d, 4 weeks then 2 or 8 h PC, 4 weeks; 30/30 (100%)	IELT (stopwatch): Week 8 tramadol daily 202.5 s, 2 or 8 h PC, 238.2 s (p < 0.001 vs baseline); placebo daily 94.8 s (p = 0.632 vs baseline) placebo 2 or 8 h PC 96.6 s (p = 0.611 vs baseline). Coital frequency tramadol daily 4.32/week (p = 0.005) tramadol 2 or 8 h PC 4.86/week (p = 0.005). Coital frequency placebo daily 2.88/week (p = 0.875) placebo 2 or 8 h PC 3.23/week (p = 0.752).	The overall AE rate was 9.8% (6.7%, and 12.4% for placebo and 100 mg tramadol respectively) ED occurred in 3.33% of men (n = 1). Vertigo was observed in 3.33% of patients (n = 2); dizziness, headache, drowsiness, and common cold were observed in 6.67% of patients (n = 2 each). There were no serious AEs.	Unclear risk - allocation method and blinded outcome assessment not reported
Kaynar 2012 [17] (Turkey) 8 weeks	IELT ≤2 min during 90% intercourse episodes All lifelong PE ED, excluded	- Tramadol 25 mg, 30/30 (100%) - Placebo, 30/30 (100%) 2 h PC	IELT (stopwatch): see Figure 3. Ability of ejaculation control (AEC): Tramadol: Mean increase 2.0 Placebo: Mean increase 0.57 Tramadol better than placebo (p < 0.001) Sexual satisfaction scores (SSS) Tramadol: Mean increase 1.80 (SD 0.98). Placebo: Mean increase 0.53 (SD 0.92) Tramadol better than placebo (p < 0.001)	Any adverse event: Tramadol: 27% Placebo: 0% Mild nausea/headache: Tramadol: 20% Mild somnolence: Tramadol: (6.5%)	Unclear risk - allocation method and blinded outcome assessment not reported
Safarinejad 2006 [19] (Iran) 8 weeks	IELT ≤2 min during 90% coitus All lifelong PE ED, excluded	- Tramadol 50 mg, 29/32 (91%) - Placebo, 28/32 (88%) 2 h PC	IELT (stopwatch): see Figure 3. IIEF: intercourse satisfaction: Tramadol: mean change 4 Placebo: mean change −1 Between-groups p < 0.05	Any adverse event: Tramadol: 28% Placebo: 16% (mainly nausea)	Unclear risk - blinded outcome assessment not reported
Xiong 2011[22] (China) 12 weeks	IELT ≤2 min All lifelong PE ED, NR	- Tramadol 50 mg 2 h PC with behavioural therapy (not reported which) (n = 36) - Behavioural therapy alone (n = 36);	IELT (stopwatch): see Figure 3. IIEF Tramadol + BT: mean change 4 BT alone: mean change 2 Between-groups p < 0.05	Any adverse event: Tramadol: 28% Placebo: 0% Tramadol: nausea (11.1%), vomiting (2.8%), dry mouth (5.6%), dizziness (8.3%).	Unclear risk - allocation method and blinded outcome assessment not reported (unable to assess fully – body text of article in Chinese)

/d, daily; DSM, Diagnostic and Statistical Manual of Mental Disorders; ED, erectile dysfunction; IELT, intra-vaginal ejaculatory latency time; IIEF, International Index of Erectile Dysfunction; NR, not reported; PC, pre-coitus; PE, premature ejaculation.

### Risk of bias assessment of RCTs

**Figure 2:**
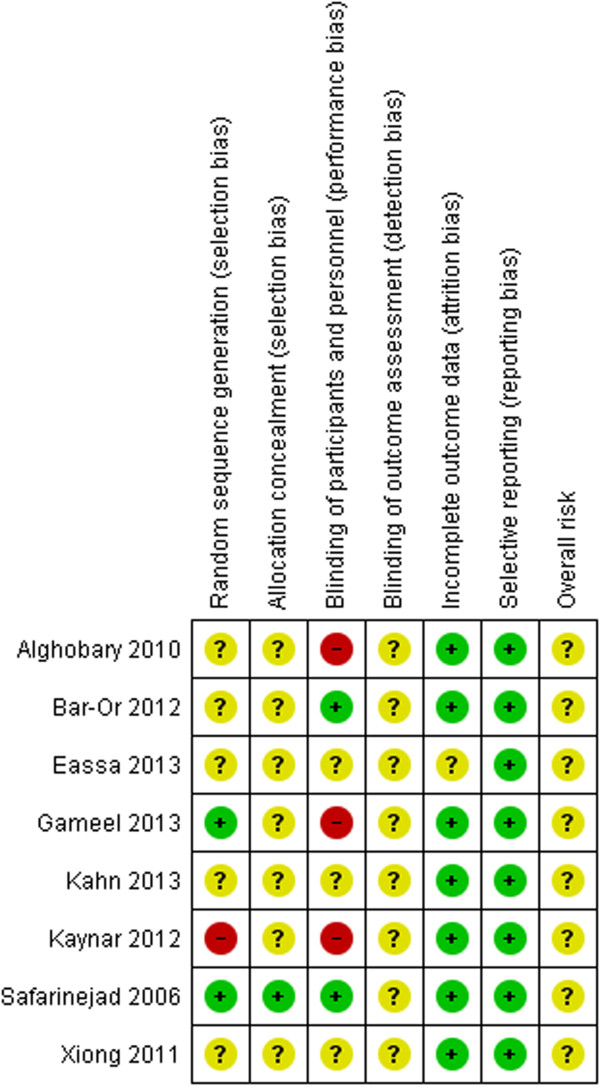
**Risk of bias assessment summary by RCT.**

The majority of RCTs were considered at overall unclear risk of bias mainly due to lack of reporting of information to inform the risk of bias assessment. Three RCTs were described as single-blind and were considered at high risk of performance bias [15–17]. One RCT was considered to be at overall high risk of bias as randomisation to study groups was according to patients’ presentation sequence at clinic, suggesting a non-random component in the sequence generation [17]. A summary of the risk of bias assessment for each included RCT is presented in Figure 2.

### Characteristics of RCTs

RCT details of the treatments, efficacy and safety outcomes, and the risk of bias assessment are presented in Table 1. Where reported, the definition of PE was varied and was defined according to: DSM-IV (Diagnostic and Statistical Manual of Mental Disorders) criteria [15, 18], an IELT of two minutes or less [16, 17, 19], or was not reported [20]. The majority of RCTs recruited samples comprising men with lifelong PE and without erectile dysfunction.

Tramadol was prescribed across all RCTs, on-demand one to four hours prior to sexual intercourse. Prescribed doses varied and range from 25 mg [17, 20] to 100 mg [20, 21]. Comparators included placebo [17–19, 21], selective serotonin re-uptake inhibitors (SSRI) [15, 16], phosphodiesterase-5 (PDE5) inhibitors [16], anaesthetic gel [16] and behavioural therapy [22]. One RCT evaluated three tramadol doses only (no placebo) [20]. Treatment duration ranged from four weeks to six months. The majority of included RCTs were 8 weeks duration. Only one trial was undertaken in the EU (across 11 EU countries) [18]. The remainder were undertaken in Egypt [15, 16, 20], Turkey [17], India [21], Iran [19] and China [22].

### Outcome data reported by RCTs

**Figure 3:**
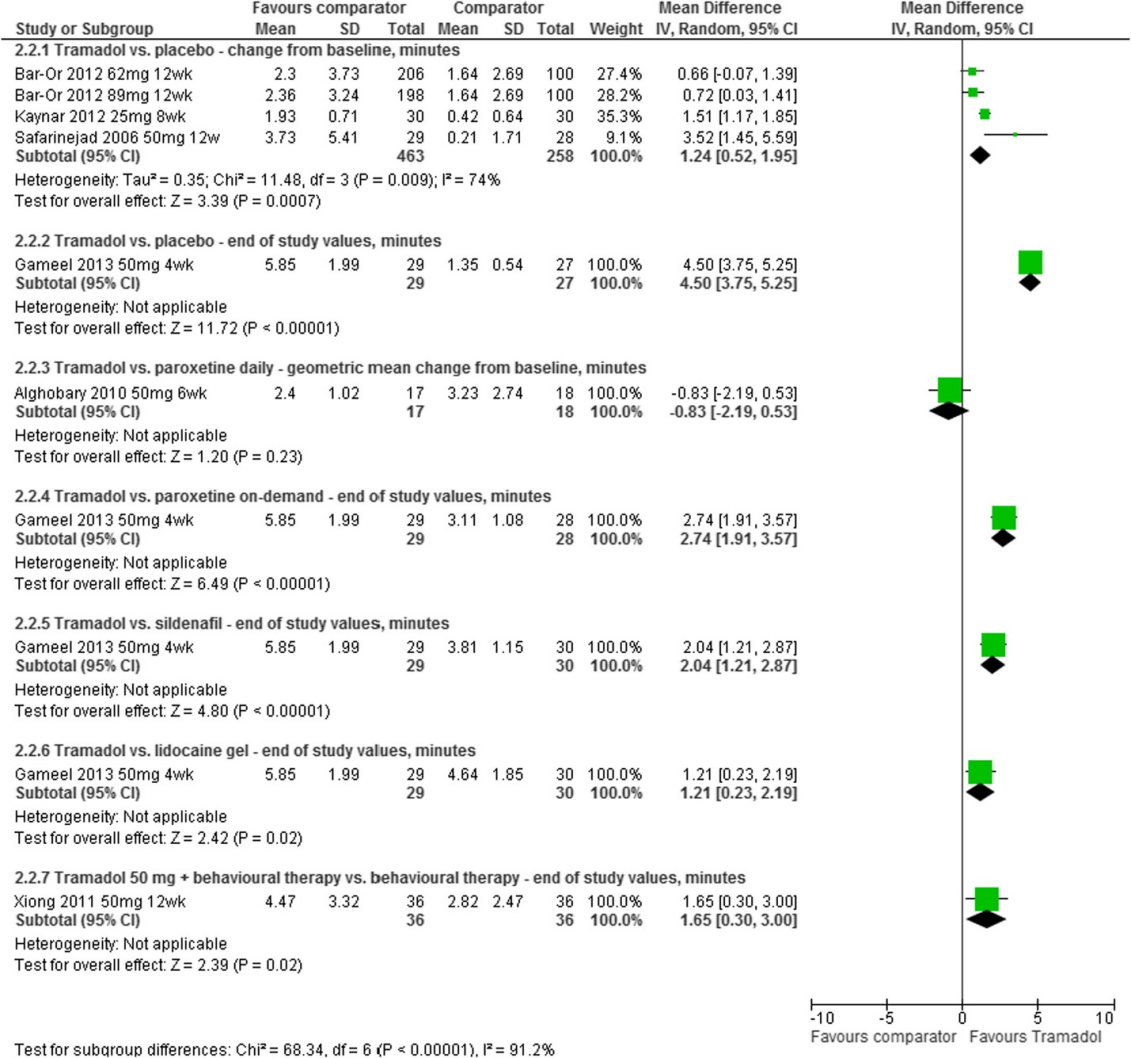
**Tramadol vs. comparator - forest plot of IELT outcomes.**

**Figure 4:**
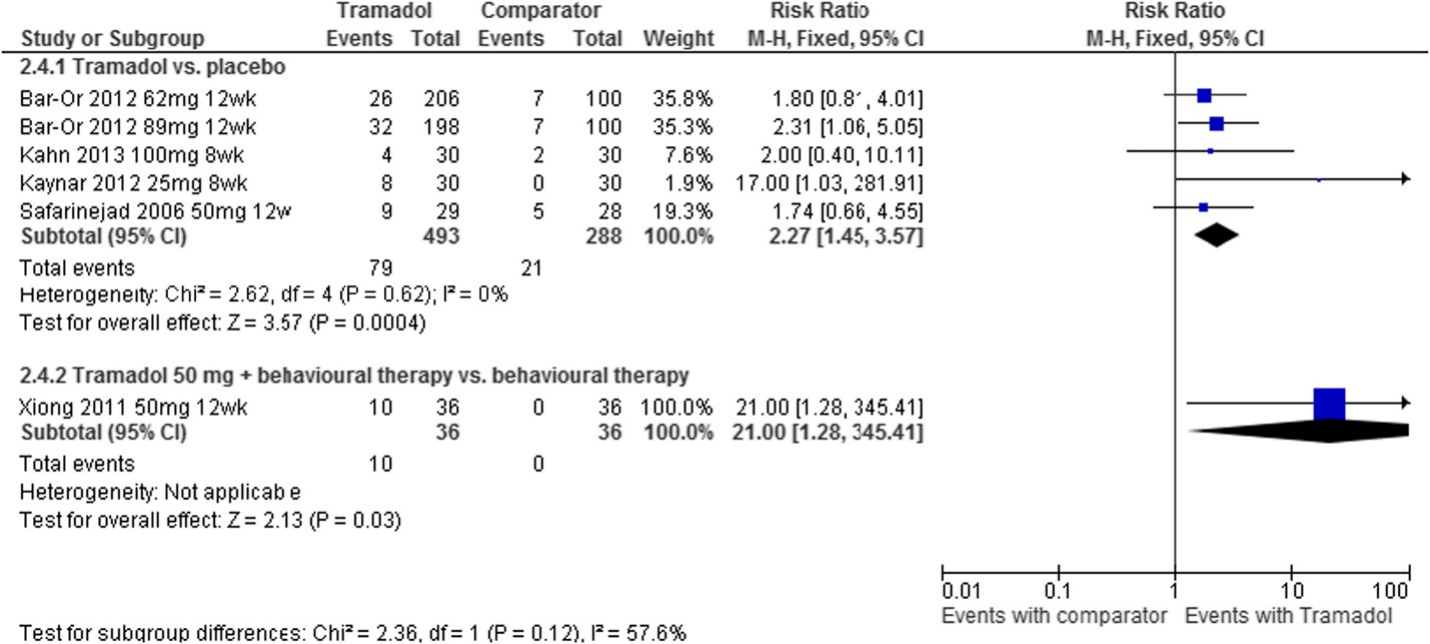
**Tramadol vs. comparator - forest plot for adverse events.**

IELT was assessed by all of the included RCTs (Table 1, Figure 3, Figure 4). Where reported, the assessment method was by stopwatch. The reporting of other efficacy outcomes was much more varied, both in the assessment method and the outcome data available (Table 1). Across the majority of RCTs, outcome data for adverse event reporting was disparate in terms of limited reporting of types of adverse events and patient numbers.

### Data synthesis

IELT as a mean outcome with a variance estimate was available for all but two RCTs [21, 23]. One reported significant changes in IELT at week eight in the tramadol group (p < 0.001) [23]. P-values for the between-group difference compared with placebo, or the change in the placebo group, were not reported. One RCT reported significant changes in IELT at week eight with tramadol daily (p < 0.001) or on-demand (p < 0.001), but not placebo (p = 0.632 and 0.611). P-values for the between-group difference were not reported.

*Tramadol vs. placebo:* Meta-analysis of mean IELT change (minutes) at 8 or 12 week follow-up, based on -four RCT study group comparisons from -three RCTs (n = 721), displayed high heterogeneity (I^2^ = 74%). The pooled mean difference (MD) in IELT was 1.24 minutes, favouring tramadol [MD (random effects) 95% confidence interval [CI], 0.52 to 1.95; p = 0.009]. The between-group difference in end of study values at four weeks based on one RCT (n = 56) was 4.50 minutes (95% CI 3.75 to 5.25; p < 0.00001), in favour of tramadol. The forest plot for these analyses is presented in Figure 2.

Significant improvements on measures of the Premature Ejaculation Profile (PEP) (p < 0.05 for all) with tramadol compared with placebo were reported by one RCT [18] Significant between-group differences on the International Index of Erectile Function (IIEF) mean number of coitus per week and mean intercourse satisfaction favouring tramadol (p < 0.05) were reported by one RCT [19] A statistically significant increase in weekly coitus associated with tramadol daily (p = 0.005) or on-demand (p = 0.005) was reported by one RCT (p-values for placebo p = 0.875 and 0.752 respectively) [21]. One RCT reported significant improvements on ability of ejaculation control and sexual satisfaction scores (instrument not reported) for tramadol over placebo (p < 0.001 for both) [17]. One RCT reported a significant between-group difference of p < 0.05 on the IIEF intercourse satisfaction score in favour of tramadol [17].

Where reported, adverse events associated with tramadol included: erectile dysfunction, constipation, nausea, headache, somnolence, dry mouth, dizziness, pruritus (itching), and vomiting. Meta-analysis of numbers experiencing adverse events at 8 or 12 week follow-up displayed low heterogeneity (I^2^ = 0%). The pooled relative risk (RR) across five RCTs (583 participants) was 2.27 [RR (fixed effect) 95% confidence interval [CI], 1.45 to 3.57; p = 0.0004] in favour of placebo (lower risk). The forest plot for this analysis is presented in Figure 4.

*Tramadol vs. paroxetine (SSRI):* The between-group difference in geometric mean IELT (minutes) at 6 weeks, based on one RCT (n = 70) comparing tramadol with paroxetine taken daily, was −0.83 [95% CI, −1.80 to 0.14; p = 0.09]. The between-group difference in end of study mean IELT (minutes) at four weeks based on one RCT (n = 57) was 2.74 (95% CI 1.91 to 3.57); p < 0.00001, in favour of tramadol compared with paroxetine taken on-demand (Figure 2).

One RCT [15] reported that paroxetine daily improved the Arabic Index of Premature Ejaculation score at 6 weeks (p < 0.05) and 12 weeks (p < 0.05) whereas tramadol improved AIPE at 6 weeks but not at 12 weeks. One RCT reported that both tramadol and paroxetine on-demand were associated with comparable improvements in sexual satisfaction (p > 0.05) [16].

One RCT reported that mild headache and gastric upset were associated with paroxetine daily and mainly gastric upset with tramadol [15] One RCT reported that sleep disturbance, dry mouth, nausea, dizziness, fatigue, vomiting, sweating, and headache were reported with tramadol, sildenafil and paroxetine on-demand, but that all side effects were tolerable [16].

*Tramadol vs. sildenafil (PDE5 inhibitor):* The between-group difference in end of study mean IELT (minutes) at four weeks based on one RCT (n = 59) was 2.01 (95% CI 1.21 to 2.87); p < 0.00001, in favour of tramadol (Figure 2).

*Tramadol with behavioural therapy vs. behavioural therapy alone:* The between-group difference in mean IELT (minutes) at 12 weeks, based on one RCT (n = 72), was 1.65, significantly favouring tramadol combined with behavioural therapy [95% CI, 0.30 to 3.00; p = 0.02]. The forest plot for this analysis is presented in Figure 2. The same RCT reported a between-group difference at 8 weeks of P < 0.05 on the International Index of Erectile Function (IIEF) favouring the tramadol group [22]. The between-group difference in numbers of participants experiencing adverse events at 12 weeks was 21.00 [RR (random effects) 95% confidence interval [CI], 1.28 to 345.410; p = 0.03] in favour of behavioural therapy alone (lower risk). The forest plot for this analysis is presented in Figure 4.

*Tramadol vs. lidocaine gel:* The between-group difference in end of study mean IELT (minutes) at four weeks based on one RCT (n = 59) was 1.21 (95% CI 0.23 to 2.17); p = 0.02, in favour of tramadol (Figure 2). The same RCT reported that tramadol was associated with significantly better sexual satisfaction scores than was the local anaesthetic (p < 0.05) [16].

**Figure 5:**
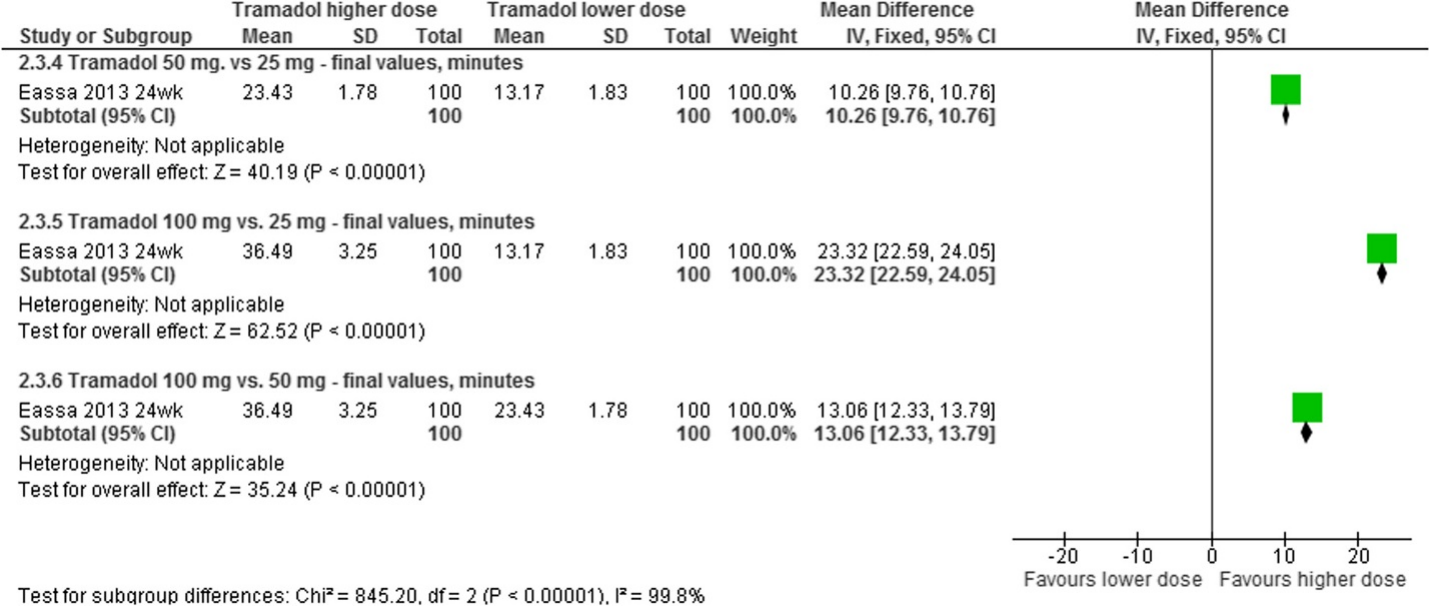
**Tramadol different doses - forest plot of IELT outcomes.**

*Tramadol 25 mg, 50 mg, or 100 mg:* One RCT (n = 300) evaluated three different doses of tramadol. The between-group differences in mean IELT (minutes) at 24 weeks were: 10.65 in favour of tramadol 50 mg *vs.* 25 mg [95% CI, 9.76 to 10.76; p < 0.00001]; 23.32 in favour of tramadol 100 mg *vs.* 25 mg [95% CI, 22.59 to 24.05; p < 0.00001]; and 13.06 in favour of tramadol 100 mg *vs.* 50 mg [95% CI, 12.33 to 13.79; p < 0.00001]. The forest plot for this analysis is presented in Figure 5. The same RCT [20], reported that all patients in the trial experienced one or more adverse events (all experienced somnolence and pruritus).

## Discussion

Pooled evidence across four RCT study groups (721 participants), suggests that tramadol is significantly more effective than placebo at increasing IELT over eight to 12 weeks. However, a high level of between-trial statistical heterogeneity is evident. The largest between-group effect size (3.52 min) was notable for one RCT [19]. Three clinical studies by the same investigator have been retracted in the past three years. However, excluding this RCT from the analysis did not significantly alter the overall effect size (1.02 min) or reduce the between-trial heterogeneity (I-squared = 71%).

The placebo-controlled RCTs prescribed tramadol doses from 25 mg to 89 mg. Reporting of the methodological quality across these RCTs was limited. Blinded outcome assessment was not reported by any of the RCTs, which may have contributed to detection bias. Allocation concealment was not reported by five of RCTs, which may have contributed to selection bias [16–18, 20–22]. One of the RCTs randomised participants according to their presentation sequence at clinic, which may have also contributed to selection bias [17]. As such, these results should be interpreted with caution.

The evidence from one RCT (70 participants) suggests that there is no difference in IELT between tramadol taken two to three hours prior to sexual intercourse and paroxetine daily [15]. Conversely, evidence from another RCT (59 participants) indicates that tramadol is significantly more effective than paroxetine taken on-demand at increasing IELT [16]. However, concealment of allocation and blinded outcome assessment were not reported by either RCT, and treatment duration was relatively short (six and four weeks respectively). As such, these results should be interpreted with caution. One of these RCTs also did not include a placebo group comparison [15]. Commonly used SSRIs in the management of PE including paroxetine (20 to 40 mg/d), are prescribed daily [9]. SSRIs such as paroxetine are absorbed slowly [24]. The half-lives of fluoxetine, paroxetine and sertraline range from 16 to 96 hours [25]. The pharmacokinetic properties of paroxetine may also account for the diverse results for the effects of tramadol compared with paroxetine on IELT.

Single RCT evidence also suggests that tramadol is significantly more effective than sildenafil, lidocaine gel, or behavioural therapy on IELT in men with PE. However, reporting of the methodological quality is limited in terms of concealment of group allocation and blinding of the outcome assessment across all RCTs included by this review.

Various assessment methods in terms of ejaculation control, patient/partners sexual satisfaction, anxiety and other patient-reported outcomes have been used across RCTs to measure the effectiveness of tramadol. Across placebo-controlled RCTs, tramadol was reported as significantly more effective than placebo for various patient-reported outcomes. Pooled evidence across trials (817 participants) suggests that tramadol is associated with significantly more adverse events including: erectile dysfunction, constipation, nausea, headache, somnolence, dry mouth, dizziness, pruritus (itching), and vomiting, than placebo or behavioural therapy over eight to 12 weeks of treatment. Addiction to tramadol by patients treated with tramadol for PE was not assessed in the current evidence base. Likewise, patient acceptability of treatment was not reported. However, one RCT reported 100% follow-up of all patients prescribed 25 mg, 50 mg or 100 mg of tramadol over 24 weeks [20]. All participants at all doses (100%) reported somnolence. The trial was considered of unclear methodological quality.

With the exception of one RCT [18], all of the included RCTs were conduction in non-EU countries, five being conducted in Middle East and Arab State countries [15–17, 19, 20]. In a population-based stopwatch study, Waldinger *et al.*[26] observed the largest difference in IELT observed between Turkey and participants from the United Kingdom and the United States. Because characteristics of PE may differ culturally, the observations from this review might not be generalizable across men from EU countries.

The risk of bias assessment indicates the majority of RCTs of tramadol in the treatment of PE are of unclear risk of detection bias, mainly due to limited reporting regarding blinding of the outcome assessment. Key aspects of best practice in RCT design to minimise bias include a robust randomisation method, concealment of treatment group allocation, and, where possible, blinding of participants and trial personnel, and blinded outcome assessment; all of which should be clearly stated in the RCT report [27].

Although our database search strategy was comprehensive, the possibility of a publication bias cannot be discounted. Insufficient numbers of RCT comparisons were available for any meaningful assessment of funnel plot symmetry to be undertaken. Nonetheless, although the RCTs identified for inclusion were of unclear methodological quality, it could be considered unlikely that any additional unpublished data for the effects of tramadol compared with placebo would contribute significantly to the overall findings of this review.

The RCTs evaluating tramadol identified for inclusion evaluated treatments over four to 12 weeks and none reported a long-term follow-up on efficacy and safety outcomes, including addiction potential. However, more important is a requirement for clearer evaluations of the relationship between treatment-related increases in IELT, ejaculatory control and sexual satisfaction associated with tramadol. Adverse event data suggest that tramadol is associated with a number of adverse events, but that these appear tolerable. However, the long-term use of tramadol for the treatment of PE in terms of a safety profile including addiction potential is unclear from the current evidence base.

The results observed by this review for the effectiveness of tramadol in treatment of PE are comparable with other reviews [6–8]. However, where meta-analyses have previously been undertaken, methodological errors are evident [7, 8]. This review has pooled data across RCTs, where appropriate, in a meta-analysis using a mean difference to summarise IELT outcomes and has avoided double-counting of participants in the analysis.

The European Association of Urology 2014 Guidelines on male sexual dysfunction recommend that pharmacological treatment options include ‘on demand’ dapoxetine, daily use of a longer acting selective serotonin reuptake inhibitor (SSRI) [off-label use], daily use of clomipramine (off-label use), ‘on demand’ topical local anaesthetic agents (off-label use) and ‘on demand’ tramadol (off-label use) [9]. Given that tramadol has been extensively evaluated against placebo for the treatment of PE in the current evidence base, with limited head-to-head comparisons between tramadol and other treatments (paroxetine, sildenafil and lidocaine gel), further direct comparisons between tramadol and other SSRIs including dapoxetine, other PDE5 inhibitors, and other topical anaesthetics should now be investigated. Whilst the observed increases in IELT were statistically significant in favour of tramadol, it is difficult to quantify how acceptable and meaningful these changes are for men with PE, without being able to evaluate the relationship between IELT, ejaculation control, and sexual satisfaction from the current RCT evidence base for tramadol. The trade-off between IELT and other effectiveness outcomes versus adverse effects and addiction potential should also be further evaluated.

## Conclusion

Tramadol appears more effective than placebo or behavioural therapy in the treatment of PE. However, these findings should be interpreted with caution given the observed levels of between-study heterogeneity and the methodological quality of the available evidence.

## Electronic supplementary material

Additional file 1: **MEDLINE search strategy.** (DOCX 13 KB)

## Abbreviations

DSM: Diagnostic and Statistical Manual of Mental Disorders
EU: European Union
IIEF: International Index of Erectile Function
IELT: Intra-vaginal ejaculatory latency time
PDE5: Phosphodiesterase-5
PE: Premature ejaculation
RCT: Randomised controlled trial
SSRI: Selective serotonin reuptake inhibitor.
