# Supplementary material for: Tramadol for premature ejaculation: a systematic review and meta-analysis
Source: BMC Urol. 2015 Jan 30;15:6. doi: 10.1186/1471-2490-15-6 (PMC4417346; doi:10.1186/1471-2490-15-6)
Supplement: Supplementary file 1 — Additional file 1: MEDLINE search strategy. (DOCX 13 KB) [file 12894_2014_409_MOESM1_ESM.docx]

**MEDLINE search strategy**

1. exp Ejaculation/

2. exp Premature Ejaculation/

3. (premature$ adj3 ejaculat$).ti,ab.

4. (early adj3 ejaculat$).ti,ab.

5. (rapid adj3 ejaculat$).ti,ab.

6. (rapid adj3 climax$).ti,ab.

7. (premature$ adj3 climax$).ti,ab.

8. (ejaculat$ adj3 pr?ecox).ti,ab.

9. or/1-8
